# A Model-Based Systematic Innovative Design for Sonic Logging Instruments in Natural Gas Wells

**DOI:** 10.3390/s24186087

**Published:** 2024-09-20

**Authors:** Chong Jiang, Wu Zhao, Miao Yu, Kai Zhang

**Affiliations:** School of Mechanical Engineering, Sichuan University, Chengdu 610065, China; 2020323025020@stu.scu.edu.cn (C.J.); zhaowu@scu.edu.cn (W.Z.); miaoyu@scu.edu.cn (M.Y.)

**Keywords:** sonic logging, conceptual design, model-based system design, fault diagnosis

## Abstract

With the continued development of natural gas extraction technologies, the accurate determination of downhole temperature and pressure has become increasingly important. It is crucial for the optimization of gas well production and an important measure to prevent accidents. However, existing logging instruments have a series of deficiencies in measurement and cannot adequately monitor modern natural gas. In response to these problems, in this paper, we propose a new model-based systematic innovation design method for designing logging instruments and simulations using finite element software. Our research results confirm the theoretical and practical utility of this model-based design method and provide a novel approach to designing logging instruments.

## 1. Introduction

With the continued development of natural gas extraction technologies, the accurate identification of downhole temperature and pressure has become increasingly important. It is crucial for the optimization of gas well production and an important means of preventing accidents. When oil pipes and other components working in the high-temperature, high-pressure, and multiphase environment of the well are corroded and perforated, accidents may occur, resulting in significant economic losses. When working with gas wells involving high-temperature, high-pressure, and corrosive media (such as sulfides), the accurate prediction of temperature and pressure is crucial in the prevention of hydrogen embrittlement and steel fracturing. Gas layer pressure and bottom hole flow pressure are parameters during natural gas extraction. That said, the instruments used to measure said parameters feature a series of shortcomings. Specifically, (1) when estimating the distribution of downhole pressure through numerical modeling by measuring wellhead pressure, data deviations are common and require several corrections; (2) sensors permanently installed in wells are at risk of failure and are difficult to replace after damage; (3) the typical problems of high temperature, scaling, waxing, and asphaltene in the well can make the operation of wire hoisting pressure gauges a risky endeavor; and (4) measurements made using optical fiber [1,2] and resistance methods [3] may encounter issues relating to sensor damage, signal attenuation, environmental interference, and forward and inverse mismatching of data.

Sonic waves have different propagation speeds between media of different densities, and echoes will, therefore, be received at different times. Taking advantage of this characteristic, acoustic wave measurement is commonly used to acquire data in the exploration of gas reservoirs [4,5], the diagnosis of faults [6], and the operation of oil and gas production [7,8]. At present, acoustic logging usually involves hanging an acoustic transmitter and receiver downhole for measurement. However, these measurement methods need to fully utilize the advantages of acoustic waves in pipelines, such as concentrated energy, low dissipation, and the ability to receive and distinguish weak signals over long distances. The effective use and arrangement of sensors can help maximize the efficiency of oil and gas wells, minimize production costs, and reduce waste [9]. In this study, we aimed to design a logging instrument that utilizes these acoustic wave characteristics and implement it at the wellhead for the purpose of measurement. In doing so, we hoped to reduce the likelihood of damage to instruments while obtaining high-quality downhole data. Logging in this way allows abnormal conditions, temperatures, and pressure gradients to be detected without stopping the well’s production, and results can be provided immediately. Designing such a logging instrument has important scientific significance and engineering value.

Currently, the field of acoustic downhole measurement is lacking an instrument-oriented design method that can effectively convert design requirements into design solutions. In the initial stage of design, that is, in the conceptual design stage, a holistic approach must be adopted to ensure that all relevant design requirements are fully considered and integrated; this may include establishing a comprehensive demand capture mechanism, performing system-level decomposition of functional behaviors, and using virtual simulation to verify the potential design results of a given design.

Model-based system engineering (MBSE) is an important aspect of digital engineering [10]. Although progress has been made by utilizing it within multi-domain unified modeling and system integration, its limitations emerge in the early stages of product design. For example, MBSE relies too much on existing paradigms in prompting designers to propose solutions, and it cannot effectively transform design requirements into design solutions. Therefore, based on the concept of MBSE, in this paper, we introduce a new design paradigm—a model-based design method that can better integrate and respond to user needs, overcome future design-related challenges, and promote innovation by ensuring the anticipation and utility of technical solutions. Currently, systematic approaches are relatively scarce throughout the design process, which, to some extent, limits innovation in and overall optimization of instruments for downhole measurement.

Much of the existing research on innovative design focuses on establishing a single design process, solving specific technical problems, and considering products from a singular perspective. In doing so, it lacks a systematic research strategy that is able to handle the design of complex products. Therefore, it is necessary to consider the requirements of complex product design from multiple perspectives in the conceptual design stage. An effective conceptual design framework will facilitate a qualitative analysis of the product at hand, which then reduces complexity and uncertainty [11].

In this paper, we propose a model-based system design method that is applicable to instruments. We chose an instrument for monitoring natural gas downhole pressure based on sonic waves as a case study. Through analysis and simulation of this case, we not only prove the feasibility of the proposed model-based system design method in practice but also demonstrate its capacity to improve the quality and innovation of design solutions. This case also presented the opportunity to create a new methodology for designing instruments.

The contributions of this study can be summarized as follows:

1. A model-based and innovative system design model is proposed;
2. A conceptual design scheme for natural gas well sonic logging instruments is constructed;
3. The efficacy of this conceptual design scheme for sonic logging instruments is then verified through finite element virtual simulation.

The structure of this paper is as follows. Section 1 is an introduction; Section 2 contains a literature review of related methods and key technologies. Section 3 describes the proposed framework. Section 4 introduces the main methods—demand transformation, model-based system design, and innovative model-based system design methods. Section 5 describes the application of the proposed framework and method in relation to natural gas downhole sonic logging instruments. Section 6 provides the conclusions of this paper.

## 2. Related Works

### 2.1. The Role of Instruments in Oil and Gas Exploration

The design of instruments takes one of two forms: improved design and completely new design. There are significant differences in their design ideas and internal logic. Improved design focuses on optimizing and improving the performance parameters of existing instruments by applying new technologies in pursuit of more accurate measurement data and stronger environmental adaptability; in the design process, emphasis is placed on existing instrument performance parameters. Some functions and structures are used as entry points for improved design, and the sensing process of the instrument is controlled either through the conversion of different forms of energy to achieve functional improvements [12] or through different optimized structural layouts to achieve better performance and prolonged service life [13]. The design of a new logging instrument must begin with a survey of the downhole in question and its production needs; the internal working logic of the instrument must be considered before a prototype of it can be produced.

In the design of such a prototype, logical design, that is, defining and optimizing the instrument’s systematic control logic and data exchange process in detail, must occur in the early stages. Logical design is fundamental to instrument design. It ensures the proper working of system functions and guarantees the stability and reliability of the system under different operating conditions. FPGA (field programmable gate array) is the most effective means of prototype design because it has a high degree of flexibility that enables rapid replication and modification of a given design during the logic design stage [14], thus providing sufficient space for innovation and enough flexibility to implement various instrument functions. That said, logical design necessitates FPGA or similar professional and powerful design tools for logical design and expression of demand, which makes it difficult for cross-domain designers to initiate. At the same time, it is not conducive to effective logical designs that benefit both designers and users. This issue might be ameliorated by abstracting the given solution, using pseudocode to express logical algorithms, and using block diagrams and flow charts to express the results of logical design. Although this method helps to simplify logical design, it may not be comprehensive; it may lack the capacity to design logging instruments, particularly those with complex functional and structural elements. A more systematic approach is therefore needed to solve this problem. Using a system engineering approach to comprehensively consider the entire system from multiple dimensions and perspectives can ensure comprehensiveness and consistency in designs. Yu et al. proposed a new systematic instrument for designing core insulation by combining the Function-Behavior-Structure (FBS) model and the Theory of Inventive Problem Solving (TRIZ) [15]. However, the authors did not comprehensively explain the differences between this instrument’s design process and other product design procedures, thereby leaving room for further exploration in subsequent research on new instrumental designs.

### 2.2. System Engineering and MBSE Methods

System engineering is a comprehensive method that integrates multiple disciplines and can deepen our understanding of the parts or all of a certain system [16]. The International Council on Systems Engineering (INCOSE) defines MBSE as “the formal application of modeling to support system requirements, design, analysis, verification, and validation activities, starting from the conceptual design stage and continuing to the development and subsequent life cycle stages”. MBSE is a digital representation of system engineering [17] and is broadly used as an important means of facilitating digital twins and physical information systems. The three major components of MBSE’s implementation include the modeling methodology, modeling tools, and modeling language. The modeling methodology establishes the connection between the modeling language and modeling tools. The foremost MBSE methodologies are Arcadia, Harmony-SE, MagicGrid, and OOSEM. Modeling tools are pieces of software that support modeling languages and perform modeling work; some examples are Capella, Rhapsody, and CatiaMagic. Modeling languages are mainly based on the Unified Modeling Language (UML), System Modeling Language (SysML), and System Modeling Language v2 (SysMLv2). However, the guidance and involvement of methodology in most current modeling languages and modeling tools are relatively limited, resulting in difficulties in developing and implementing MBSE.

The MBSE metamodel is a complex model framework that accounts for abstract syntax in the modeling language and is used to comprehensively model the system during its development lifecycle. The MBSE metamodel provides semantic mapping—from the domain ontology to the system—by defining the kinds of objects existing in the system, the relationships between object types, the attributes of object types, and the rules that govern combinations of objects and relationships. The metamodel is the basis of the metamodel ontology then used to define the domain ontology [18]. It is the basis of the modeling methodology, modeling tools, and modeling language of MBSE and provides a unified perspective on and strategy for MBSE. The main advantages of the MBSE metamodel are its comprehensiveness and meticulousness. However, it also has drawbacks, such as high complexity, challenging construction, and difficulty of operation for uninitiated individuals.

The Arcadia method is a model-centric system engineering method, and its metamodel includes four levels: functional, logical, physical, and requirements. The Arcadia metamodel enables designers to analyze and design systems from different perspectives and abstract levels through the detailed division of these levels. However, Arcadia’s metamodel lacks a scientific method through which we might consider mapping requirements to functions and subsequent structures from a conceptual design perspective. Secondly, its linear hierarchical division introduces certain processing limitations. When dealing with cross-level relationships and dynamic changes, its linear hierarchical division cannot fully adapt to the design changes generated during the design process.

During the current MBSE process, the construction of models is mainly based on the precedent set by existing MBSE cases. Different designers will have different design experiences and knowledge in fields aligning with a brand-new object. The final system models they construct will differ significantly. This difference will eventually lead to multiple adjustments or major modifications within the subsequent product design process. The Arcadia method implicitly defines a modeling language and includes a modeling methodology [19]. When combined with the dedicated Capella tool, it is no longer necessary to master the development process. Using the Arcadia method to build an MBSE model involves four steps. Considering each level’s criteria, a complete systemic model is built, satisfying the requirements of operational analysis, systemic analysis, logical architecture, and physical architecture. This makes both analysis and design more systematic and organized and ensures the integrity and consistency of the design process.

### 2.3. Evolution of Conceptual Design Methods

Since Pahl and Beitz et al. first introduced conceptual design in 1984 [20], the paradigm of the product design process has changed. Ranging from functional analysis to structural solutions, the conceptual design process is complex, and each of its components is closely linked. Various designs’ demands function as the input, and satisfactory solutions function as the output; to produce said output, several iterations will be created within the design process until satisfactory results are obtained. During the continuous evolution of conceptual design, many methods have been utilized by enterprises and researchers, such as FBS [21], TRIZ, AD (axiomatic design) [22], and GDT (generic design theory) [23]. These methods abide by the basic laws of conceptual design and its most important features: functionality, knowledge, and conflict. These methods have achieved remarkable results in some specific product design fields, but they show certain limitations when dealing with complex and changing user needs. Particularly in the field of instrument product design, these traditional methods often cannot effectively integrate and respond to diverse needs, making it difficult to achieve adaptability and interdisciplinary collaboration.

As a design method that is still in the stage of continuous improvement and development [24], FBS does have flaws, such as limited applicability to different fields and uncomprehensive design knowledge. However, it is undeniable that to solve the uncertainty problems in the conceptual design process, the FBS method is still in a period of continuous improvement and updating. Combining the FBS method with other design methods and tools or using its mapping mechanism to construct. It is the main means of improving the FBS method today. Based on FBS, we may create multi-layer knowledge graphs [25] and utilize knowledge graphs to solve multi-domain knowledge conflicts within conceptual design, thereby improving the efficiency and reliability of subsequent designs.

Layering within the FBS method reflects layering within system engineering more broadly, and both emphasize interaction and coordination between different levels of the design process. This layered way of thinking is particularly critical in the early stages of product design. It is necessary to determine the broad direction in which future product development will move while reducing reliance on personal experience. Martin et al. used MBSE and systemic thinking methods to better understand the variables and factors that affect the design team [26]. Based on AD theory, Wang et al. constructed a model-based systemic design method, incorporating an information reuse framework, semantic modeling, and guidelines for reasoning [27,28,29]. Building upon this work, Hu et al. constructed a function–behavior–structure mapping model for “Internet+” [30]. Based on model-driven engineering, the FBS model was integrated with the knowledge model, and “requirement R” was introduced via ontology and SysML to establish the RFBS model [31]. The authors utilized a model-based system of knowledge alongside an ontology library and FPBS to optimize their mechanical product design protocols [32].

The aforementioned related research demonstrates that although both FBS and MBSE incorporate layering and mutual mapping between different layers, research on how we might optimally combine these two methods is scarce.

## 3. A Framework for Using the Model-Based System Design Method in Instrumental Design

**Figure 1:**
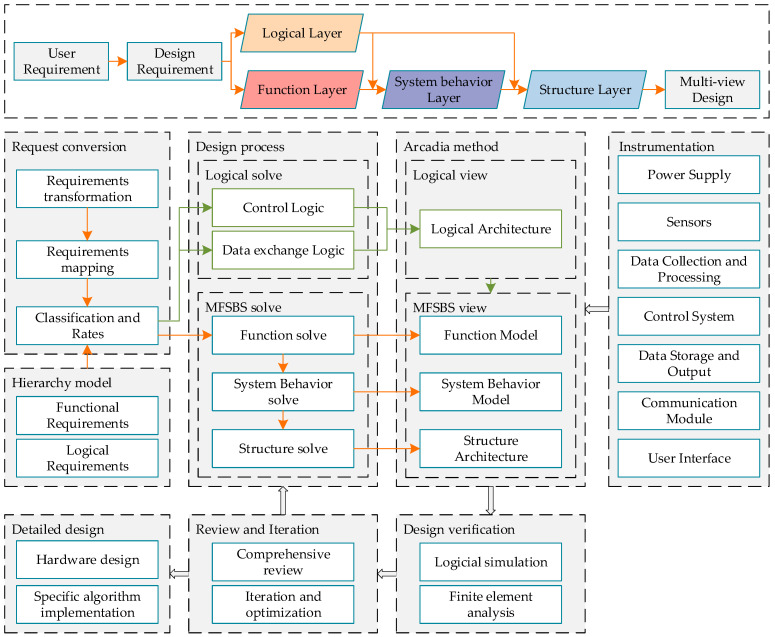
A framework for using a model-based systemic design method in instrumental design.

The core purpose of this study is to present a model-based system of innovative design that can be used to create instruments. The framework of the proposed model is shown in Figure 1.

This design framework is the basis of the entire modeling system outlined herein. It is an extension and repurposing of the Arcadia method and the FBS method. It stores, uses, and manages results through semantics and graphs in order to improve the shortcomings of existing systems and represents a paradigm shift in model-based design. Its methodological flow begins with demand conversion, following which the functional layer, system behavior layer, logic layer, and structural layer are traversed, and a multi-perspective design scheme is produced. In the figure, logical design flows are represented using green lines and other design flows are represented using orange arrows. The conversion of user needs into design requirements is the first step in this process. Subsequently, demand mapping is performed, and the design requirements are classified and sorted using a hierarchical model. Logical requirements (such as control logic and data exchange logic) are then identified, and logical solutions are introduced in response. Model-based functional, behavioral, and structural solutions are also proposed. Using the Arcadia method and the MFSBS (model-based function system-behavior structure) method, four different designs of a prototypical instrument—including its logical architecture, functional model, system behavior model, and structural architecture—are defined, and these constitute a multi-perspective scheme of instrumental design. The specific prototypical instrument includes a power supply, sensor, data acquisition and processing module, control system, data storage and output component, communication module, and user interface. The design is verified through logical simulation and finite element analysis before being comprehensively reviewed, reiterated, and optimized based on the simulation results. The final result is a detailed instrumental design that includes guidelines on both the hardware and specific algorithm involved.

## 4. Methodology

### 4.1. Analysis of Requirements

The conceptual design of the prototypical instrument must begin with an acknowledgment of fuzzy requirements. Fuzzy requirements refer to situations in which users’ descriptions of their requirements are not clear, detailed, or specific enough at the beginning of the design process, which makes the desired design difficult to understand and visualize. Fuzzy requirements have a variety of causes, such as insufficient communication, insufficient understanding of target users, and unclear design scope, and they may lead to delays in development, increased costs, poor quality, and even failure to develop a prototypical instrument. Therefore, designers need to actively communicate with users during the design process in order to understand their requirements. They must also explore fuzzy requirements that may have remained hidden, determine user requirements qualitatively and quantitatively, ensure the accuracy and clarity of user requirements, and fully understand the direction in which the project is moving. At the same time, it is necessary that designers determine the parameters within which they are working. Accordingly, the aforementioned user requirements are sorted and weighed, and the relative importance of each requirement is clarified in order to reasonably allocate resources. To effectively acquire and analyze the requisite features of a given instrument, designers must identify, transform, and classify user requirements.

#### 4.1.1. Identifying User Requirements

Identifying user requirements is an important task that designers must complete before designing a prototypical instrument. It is the basis of and key to their subsequent design and ensures a solution that will satisfy users. User requirements are often implicit and vague, so it is necessary to survey user requirements in a variety of ways (discussion with users, questionnaires, and observation of users’ behavior with similar instruments). Through these methods, we can understand user requirements, analyze their expectations of prototypical instruments, and design prototypical instruments that better meet such requirements. When surveying potential users, it is also necessary to integrate, screen, and analyze their needs and expectations; this extracts accurate and valuable data that can then be grouped into a user requirements set that we will designate UR. This dataset then provides strong support for subsequent design work. However, due to disparity in users’ knowledge and understanding of prototypical instruments, there are often clear discrepancies between the users’ understanding of requirements and the designer’s understanding of requirements. Therefore, after user requirements have been identified, they must be converted into design requirements.

#### 4.1.2. Transforming User Requirements

The purpose of this step is to reasonably and visually represent the designer’s understanding of users’ requirements and eliminate design elements that may not meet users’ expectations. After classifying the user requirements set UR, based on their own understanding, the designer obtains a preliminary design requirement set DR. One requirement in the user requirement set may correspond to multiple requirements in the design requirement set. Conversely, multiple requirements in the user requirement set may also correspond to one requirement in the design requirement set. Subsequently, the elements in the user requirement set, UR, are denoted $UR_{i}$, and the elements in the design requirement dataset, DR, are denoted $DR_{j}$. The mapping function *f* is used to represent this relationship:

$$f:{UR}_{i}\to 2^{DR}$$

**Figure 2:**
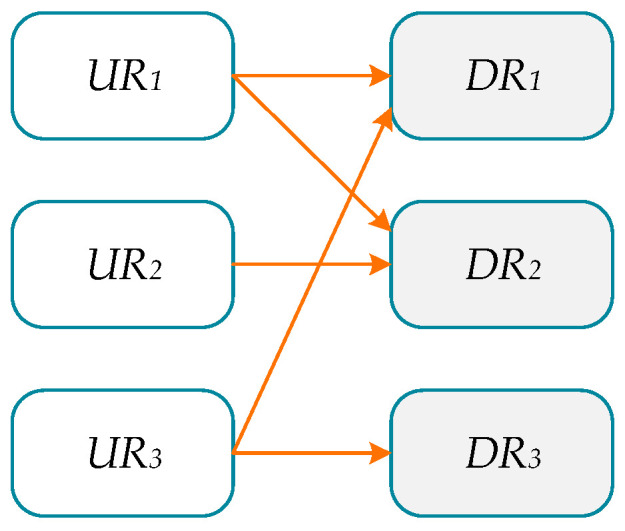
An example of a mapping diagram from user requirements to design requirements.

That is, $UR_{i}$ is mapped to a subset to DR, and $2^{DR}$ represents the power set of ${DR}_{s}$, the set of all possible subsets in DR. In this mapping relationship, a user requirement $UR_{i}$ may be mapped to multiple design requirements $DR_{j}$, and multiple user requirements $UR_{i}$ may also be mapped to the same design requirement $DR_{j}$. to intuitively display the mapping relationship between user requirements and design requirements. To facilitate communication between users and designers, the above mapping function *f* is converted into a graph that expresses mapping relationships, as shown in Figure 2.

Within this form of graph mapping, user requirements $UR_{i}$ and design requirements $DR_{j}$ are regarded as vertices in the graph and are listed on the left and right sides. The directly mapped connections between them represent the relationship between user requirements and design requirements, i.e., from a certain user requirement to one or more design requirements. In the form of a graph, designers can communicate with users intuitively, discover any conflicts in their understanding of user requirements and expectations, and augment the resulting design requirements in a timely manner.

#### 4.1.3. Classification of Requirements

**Figure 3:**
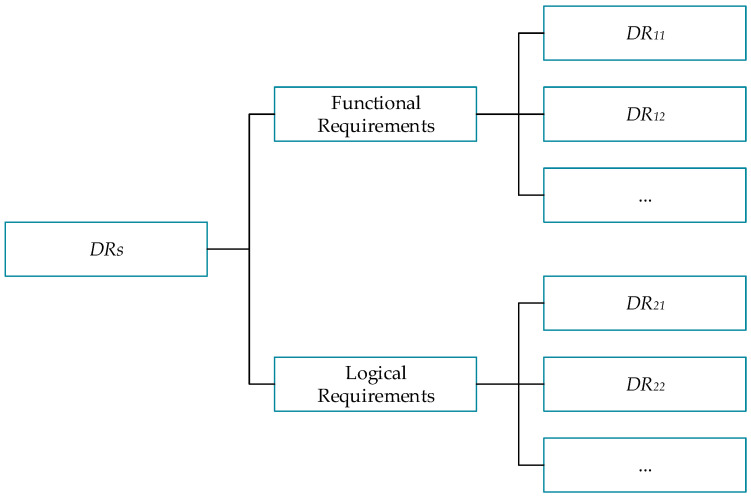
Hierarchy of design requirements.

Different designers have different design resources, so the order in which they meet design requirements will be different. To solve this problem, a method for evaluating design requirements is needed to help understand the priority judgments of different designers when facing various design requirements so as to carry out targeted resource allocation and task arrangement. In addition, different types of design requirements are guided into different design paths: for functional requirements, function mapping will be carried out and enter the function solution process; for logical requirements, they will enter the logic design process. For different types of design requirements, these requirements need to be further analyzed and evaluated to determine the category of requirements, which requirements should be implemented first, which requirements can be implemented within the current design cycle, and which requirements have a lower priority or may exceed the design scope. To obtain this division and evaluation process, it is a very efficient and intuitive processing method to use the analytic hierarchy process (AHP) to construct a design requirement hierarchy model to divide and evaluate requirements. In the analytic hierarchy process, it is first necessary to determine the structure of the analyzed object, divide the analyzed object into different levels and factors, and then compare them two by two. According to certain judgment criteria and weights, the importance of comparison and the weight relationship between each analyzed object is determined. Finally, the importance and ranking of each analysis object are obtained through the calculation formula, thus finally obtaining the analysis result. Finally, we can assign importance to each object using a formula that produces reliable results. The design requirement hierarchy model in Figure 3 illustrates their classification by importance and relevance.

The design requirement hierarchy is divided into three layers: the first layer contains all current design requirements (*DRs*), the second layer contains functional requirements and logical requirements, and the third layer contains required items separated by type. First, the functional or logical requirements are hierarchically divided according to their importance, feasibility, urgency, and other factors, and the comparisons between and weight distributions of each level are calculated and analyzed. Next, the judgment matrix $A={\left(a_{2i}\right)}_{n\times n}$ is constructed, where i corresponds to the number of items in the third level of the hierarchy. The relative importance of the different design requirements is calculated by assigning values to $a_{2i}$, and finally, the overall weight of the third level can be determined. These weights represent the relative importance of each design requirement within the entire decision-making structure.

To better understand the specific utility of these classification and evaluation steps, the specific definition and processing of functional requirements and logical requirements will be explained in detail.

In instrumental design, functional requirements may include the following: data acquisition, which entails finding the main functions that the instrument needs to complete (including obtaining and recording relevant parameters and information); signal acquisition, which entails recording signals from the environment or the object being measured, ensuring the accuracy and integrity of the data; and physical measurement, which entails measuring specific physical parameters through sensors and other equipment. After that, the functional requirements are mapped into the functional solution process, and the FBS model, under the guidance of MBSE, is used in parallel with logical design.

An instrument’s logical requirements are as follows: logical control, which refers to the strategy and logic of the instrument under different operating conditions; data processing, which refers to data analysis, calculation, and storage, ensuring the accuracy and efficiency of data processing; and processing and analysis of input signals to ensure the validity and accuracy of signals. After identifying logical constraints, the requirements are input into the logical design process. Logical requirements are specifically defined and designed at the logic layer. The design of the logic layer will ensure that the instrument maintains consistency and efficiency while fulfilling the logical requirements.

### 4.2. Model-Based Systematic Innovative Design Process

In this section, the process of designing the model-based system will be described in detail. Using MBSE guidelines, this design process involves innovative methods to fulfill functional requirements, designs controlling logic and data exchange logic for logical requirements, and finally provides a parallel design of a prototypical instrument, ensuring a multi-dimensional analysis of and practical solutions to design requirements. In accordance with the Arcadia method, our results are abstractly mapped in the model-based diagram, creating a multi-perspective conceptual design scheme for the prototypical instrument. Before further describing the design process in detail, it is necessary to explain the concept of the MFSBS model. The MFSBS model is an extension of the FBS model based on the MBSE concept, which aims to better integrate the functional, behavioral, and structural levels and provide a comprehensive design solution through a multi-perspective approach. The model contains three levels, namely the functional layer, the system behavioral layer, and the structural layer. The entire design solution is initiated through layer-by-layer mapping.

#### 4.2.1. Logical Solution Process

**Figure 4:**
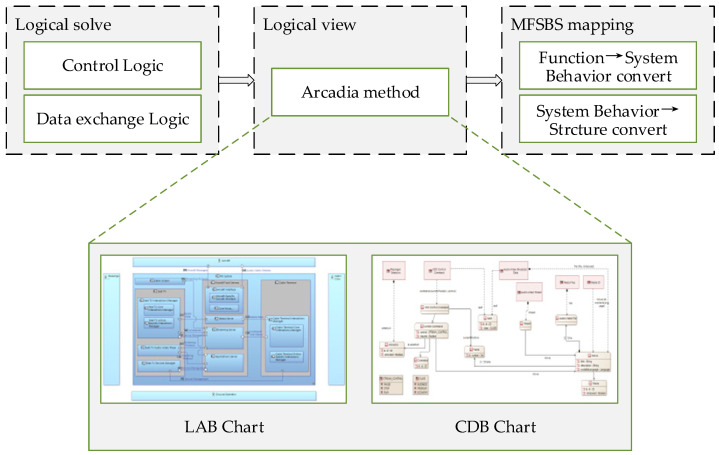
Logical solution process.

In the design of prototypical instruments, the control and data exchange of the product in the conceptual design stage informs the main testing and measurement of the instrument. After these factors have been designed and modeled, the system’s requirements and functions are allocated based on the logical architecture of the model in order to evaluate the design scheme in the early stages of the prototypical instrument’s conceptual design. The logical design process parallels other design processes because the data flow between the control and the instrument distinguishes instruments from other types of products. The instrument should be tested via measurement and reception tests performed on other objects. The capacity for control and data interaction is more important than the structure of the instrument itself. Therefore, when designing the functional structure of the instrument, the logic of the prototypical instrument needs to be designed in parallel. In addition, through such a parallel design process, the function and structure of the prototypical instrument can be controlled as logical constraints in the design process; this means that the function and structure of the instrument can better fulfill the most important design requirements, as determined by testing and measurement of the instrument. The logical solution process is shown in Figure 4.

In the logical design process, it is crucial to carry out the effective conceptual design of the instrument’s control logic and data exchange logic based on a preset instrument sensing method determined by the product’s logical design requirements. Currently, there is no need for complex and detailed designs such as logical algorithms. The main aim at this stage is to effectively and clearly display the conceptual results of the logical design. We refer to and use the construction methods and models of the logical architecture layer outlined in the Arcadia method; these include but are not limited to LAB (logical architecture blank) diagrams and CDB (class diagram blank) diagrams. As a result, the logical design results can be expressed in a unified and standardized manner; this enables designers in different professional fields to communicate with each other in a universal language and to accurately convert logical design requirements into logical design solutions. It is also a convenient means of displaying solutions to users when they come to evaluate, verify, and reiterate the design. At the same time, it will constrain the two mapping processes (from the functional layer to the systemic behavioral layer and from the systemic behavioral layer to the structure) in the subsequent design process so that the design’s features more wholly fulfill key design requirements.

#### 4.2.2. MFSBS Solve Process

**Figure 5:**
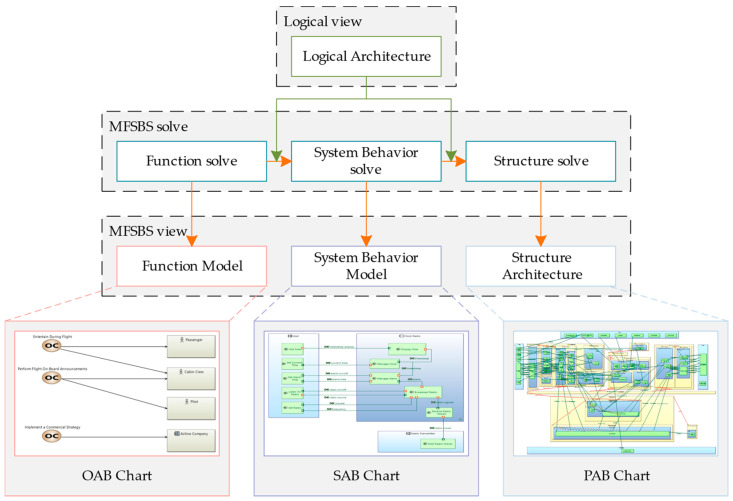
MFSBS Solution Process.

Before using MFSBS to solve functional requirements, the HoQ (House of Quality) model maps and converts the pre-defined functional design requirements using product functions. Specifically, HoQ forms the basis of the QFD (quality function deployment) method. Using HoQ to build a functional matrix, the design requirements are correlated with the technical characteristics of the prototypical instrument to determine which technical characteristics are critical to meeting customer needs. Designers can more clearly understand how to transform functional design requirements into specific product functions and consider the various factors influencing the design and development process. Using the HoQ model to implement functional requirements, transformation mapping means that users’ needs are more effectively fulfilled and development costs are reduced to a certain extent; this is because transformation mapping can help designers to more rationally allocate resources in the conceptual design stage and determine the elements of the design that need to be prioritized. It also helps designers to convert requirements into functions. According to the evaluation results, designers may need to adjust their plan, allocation of resources, and strategy to ensure the smooth execution of the design. Continuous iteration and optimization are essential during the construction and evaluation of the House of Quality model. Designers need to pay close attention to the progress of the project, compile feedback, and continuously improve and adjust the HoQ model. In this way, we can ensure that said model provides a strong and stable foundation for the success of projects and ultimately contributes to the production of high-quality and high-performance products. The MFSBS solution is shown in Figure 5.

The MFSBS model encompasses three main components: the functional solution, systemic behavioral solution, and structural solution. The results derived from these three steps correspond to the operational analysis layer, systemic analysis layer, and physical architecture layer from the Arcadia method. The logical design results provide important constraints for the two key mapping processes in the MFSBS model (function to system behavior and system behavior to structure). Each layer is converted into MBSE diagrams to unify the expression of all results, and by incorporating the results of the logical layer, we can construct a systematic, innovative design solution that integrates four different perspectives.

(1) Function layer

When working with the MFSBS model, the first thing to be constructed is the function layer. The function layer results from the conversion of the design requirements from the previous step. The model displays all functions that serve to establish the behavior layer and system architecture. Therefore, within the MFSBS model, solving the function in the first layer helps designers to think rationally about the design of the product, meaning they act in accordance with conceptual design principles, moving from abstract to concrete thinking. In the Arcadia method, the model starts with an operational analysis and then defines the goals that users wish to achieve in their work or tasks. During this step, there is no need to consider how the operation is implemented, nor is there any need to consider how the method might be applied to the system [19]. This step emphasizes starting from the user’s perspective, analyzing the goals that the user requirements to achieve in the work or tasks, and ensuring that the system design fulfills the user’s requirements and expectations. In the subsequent process, it is necessary to build systemic functions, logical functions, and physical functions at the same time. However, the existence of repeated functions at multiple levels may lead to fewer functional differences throughout the whole design process, making it difficult for designers to think about the functional differences in each step. Therefore, the functional layer—combined with functions from the FBS method—can effectively guide the functional design of a prototypical instrument and map its results to diagrams (such as the OAB (operation architecture blank) diagram), mirroring the operational analysis layer to achieve a standardized illustration of a functional design scheme.

(2) System Behavior layer

When solving problems using the FBS method, behavior serves as a bridge between function and structure, changing the focus of the original mapping method from product function to product structure. However, in the evolution of the FBS method, different scholars have given different definitions of behavior; these include how the structure of an artifact realizes its function [33], the behavior or process of an artifact in a specific natural environment [34]; the attributes derived from the structure or expected structure [21], and the physical configuration of an artifact [35]. Though these definitions differ, they all attempt to clarify the relationship between function and structure. The core purpose of constructing the behavior layer is to assist designers in designing an effective structure that aligns with the product’s function. This not only achieves good product functioning but also reduces the distance between function and structure in the forward design process. At the same time, it also helps to reduce any uncertainty surrounding the forthcoming design process and improves the reliability and efficiency of the design. However, in the actual design process, the behavior layer is not directly reflected in the final product. The aforementioned behavior usually manifests in the material in the form of attributes and definitions, resulting in implicit knowledge, which cannot be accurately valued and is difficult to fully utilize in product design. Due to these limitations, a trend is emerging in the design community: designers are gradually abandoning the behavior layer in the FBS model and returning to the more concise and direct FS (function–structure) model [36]. Therefore, in order to give full play to the importance of the behavior layer in the design process, this paper combines the behavior layer with the system conceptual framework in the model-based system innovation design method and uses the behavior and product functions obtained in the previous step. Describing the behavior of the product from a system perspective can be directly mapped to the initial design requirements, thereby ensuring that each decision result in the design process is closely related to user needs, enhancing the purposefulness and accuracy of the design. This method not only improves the systematic nature of the design but also brings higher innovation and practicality to product design. In the Arcadia method, the results of the constructed operational analysis need to be converted into system analysis for further system design, but how to perform this conversion has not been fully explained, resulting in the need for system analysis to rely on design experience and existing cases for reference. For the construction of the system behavior layer, it is necessary to use the SAB (system architecture blank) diagram and other charts in the Arcadia method to fully represent the system behavior of the entire prototype instrument and realize the mapping of function to system behavior.

Due to the varying levels of experience and knowledge of different designers, functions mapped using behavioral knowledge may have various impacts on users because of their different implementation methods. For example, multiple measurement techniques may achieve the same goal, but they may also alter the efficiency, reliability, etc., of the instrument in different ways. Therefore, logical design results should be utilized to constrain the mapping of functions to the system behavior layer; this will result in appropriate systemic behavioral design and reduce the difficulty of mapping. The system behavior layer describes the behavior of a prototypical instrument under different operating conditions, as well as the relationships and interactions between each behavior. The system behavior layer is not only an important part of the prototypical instrument’s development; it is also an important mediator of the transition from function to behavior. In addition to the existing diagram of the system’s architecture, designers may incorporate alternative diagrams based on the same architectural model to facilitate further collaboration. In doing so, they can ensure that each decision fulfills a certain design requirement and the broader design goals of the prototypical instrument.

(3) Structure layer

Within the Arcadia method, the physical architecture level’s purpose is to build physical functions and other parts of the system, which is the equivalent of designing the structures, components, and other physical objects required by the system in the real world. Moreover, within the Arcadia method, the physical architecture is designed after the logical architecture is built. Therefore, the structural layer built via the MFSBS method may have the capacity both to map behavior to structure (as in the FBS method) and to create physical architecture from logical architecture.

In the MFSBS method, the structural layer is the last step in the conceptual design of the prototypical instrument and is also the final stage in the process of transforming the fuzzy to the precise and the simple to the detailed. Structural design is a complex and critical process; use the PAB (physical architecture blank) diagram in Arcadia to analyze the structural design results. It not only involves the construction of the overall architecture but also accounts for the relationships between and optimization of each component. Whether developing a new instrument or improving an existing instrument, it is usually necessary to optimize the latter’s design or augment it in response to a large number of existing structural cases. A modular design method is suitable for this phase. From existing structural cases, parameters, shapes, and other features are matched and combined to achieve structural configuration. This procedure can produce structural features with strong universality, making it easier for the results of the novel design to interact with other models and be transformed [30]. Through modular design, complex structures can be decomposed into multiple functional modules, which can be independently designed, optimized, verified, and finally combined into a single complete system structure. This method can greatly improve efficiency and flexibility and reduce complexity. For example, by referring to and drawing on experience in designing existing sensors, actuators, and other components, we may construct and optimize a structural model that meets user requirements.

### 4.3. Design Verification

After the design of the prototypical instrument and integration of four perspectives are complete, in order to determine the feasibility of the conceptual design scheme in a timely manner, predict the performance of the prototypical instrument, and enhance designers’ confidence in the decision-making capacity of the scheme, further verification is necessary. In this phase, various pieces of software should be used to simulate the application of the design in multiple contexts and provide a basis for subsequent designs. Data simulation software such as Simulink and LabVIEW can be used to simulate the data handling and logical design of the virtual instrument. This simulation software can effectively emulate the data processing and signal processing capacity of the prototypical instrument to verify its accuracy and efficiency. Through these simulation methods, the performance of the instrument can be comprehensively evaluated, and potential problems can be identified and corrected during the conceptual design phase. Finite element simulation software such as COMSOL and ANSYS can be used to verify the functional structure of the prototypical instrument. This software can simulate the performance of the product under actual working conditions and evaluate its response and behavior under different environments, thereby accomplishing early verification of the product’s structural design during the conceptual design stage. The simulation of these two aspects can ensure the reliability and accuracy of the modeled system, effectively support subsequent iterations of the design, and thus improve its overall quality.

### 4.4. Review and Iteration

After the simulation and verification of the design scheme are complete, the results need to be comprehensively evaluated in order to produce improved iterations of the prototypical instrument. Evaluation of the conceptual design scheme should include a detailed evaluation of various elements (the logic layer, functional layer, system behavior layer, and structural layer) to ensure that each component fulfills user demands and expectations. The evaluation and reiteration of the design need to consider the following aspects. The first is logical layer evaluation; the logical design must fulfill the control-related requirements of the system and effectively process data and signals. The second is functional layer evaluation; the completeness and accuracy of the mapping transformation of functional requirements must be verified to ensure that the instrument can complete its expected operations and tasks. The third is systemic behavioral layer evaluation; the behavior of the system must be assessed under different operating conditions to ensure that its results are consistent with the functional layer. The last is structural layer evaluation; the rationality of the structural design must be ensured, along with its good manufacturability and maintainability.

When a design that fulfills requirements and user expectations has been created, the detailed stage of the prototypical instrument’s design can begin. Through this evaluation and iteration process, the quality and reliability of the prototypical instrument can be effectively improved, providing a solid foundation for subsequent detailed design and manufacturing and ensuring that the final instrument is of a satisfactory standard.

## 5. Case Study

In order to verify the effectiveness of the proposed modeling system, we selected a natural gas downhole sonic logging instrument as a case study. The aforementioned conceptual design and simulation of the instrument have demonstrated how it can be applied to the systematic design of complex instruments.

### 5.1. Case Introduction

This case study focuses on the development of a prototypical instrument that uses sonic waves to test and measure the production status of natural gas downhole. The instrument can interpret and analyze sonic waves to monitor liquid level depth, identify large changes in the cross-section of the wellbore structure, and measure pressure gradients, gas volume, and quality. We anticipated that abnormal conditions and pressure gradients would be detectable without well intervention and that test measurement results would produce immediate results; this was expected to reduce well-logging costs and potential accidents.

### 5.2. Analysis of Requirements and Conversion for Sonic Logging

**Figure 6:**
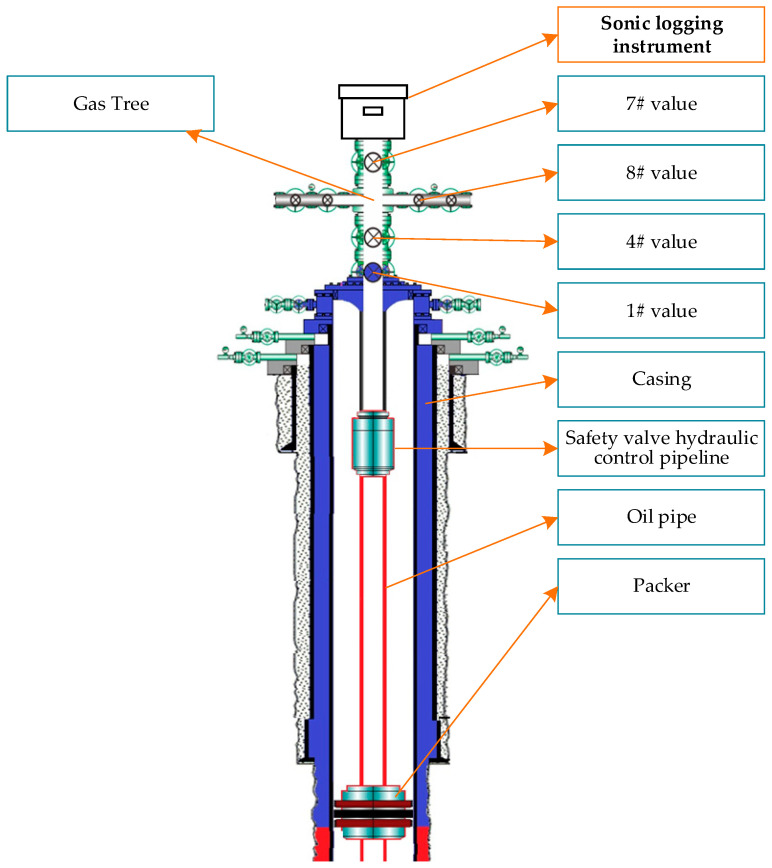
Installation position of the sonic logging instrument.

According to users’ description of the sonic logging instrument, users must connect the sonic logging instrument to the #7 valve position of the natural gas tree, open the #1 and #4 valve positions, and control the valve opening at the #8 position to measure sonic signals under conditions with various levels of oil pipe pressure. In this context, the symbol ‘#’ is used to denote valve numbering (e.g., ‘#1 valve’ refers to valve number 1). The instrument installation position is shown in Figure 6.

First, through the method of demand analysis and transformation proposed in this paper, the user’s expectations and needs are analyzed, and four user needs are defined.

$UR_{1}$: The temperature and pressure status of the well can be monitored at any time without well repair;

$UR_{2}$: The well environment can be predicted and monitored with high accuracy and high reliability, including changes in temperature and pressure;

$UR_{3}$: operation remains simple, and data interpretation is intuitive so that field personnel can quickly understand and carry out operations.

$UR_{4}$: The equipment’s maintenance is simple, ensuring long-term stable operation and reducing unnecessary downtime.

Based on the above-defined user needs, according to the actual design resources and conditions, the users’ needs are transformed into the following design requirements.

$DR_{1}$: The instrument should have the function of accurate acoustic wave transmission and reception and be able to work stably in high-temperature and high-pressure multiphase environments.

$DR_{2}$: The instrument needs to have advanced data-processing capabilities and be able to accurately calculate the temperature and pressure of the well; it should also check for the presence of corrosive gases through acoustic wave signals.

$DR_{3}$: The instrument and the instrument interface should be user-friendly and easy to operate; it should intuitively display data and provide easy-to-understand operation guidance and data interpretation.

$DR_{4}$: The instrument’s requirements include durability and maintainability, which should ensure that the equipment can operate for a long time under extreme conditions and is easy to maintain and repair.

$DR_{5}$: The instrument should have real-time data transmission capabilities, including wireless transmission capabilities, to achieve real-time monitoring and alarms.

$DR_{6}$: The instrument should be designed so that it can be installed and debugged without large-scale downhole operations; this will reduce operating costs and time.

**Figure 7:**
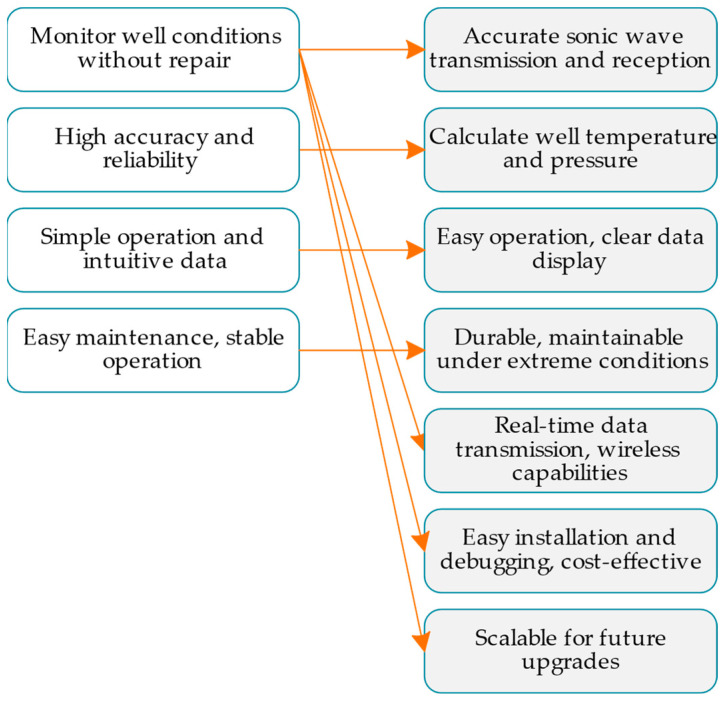
Conversion of user requirements to design requirements for a sonic logging instrument.

$DR_{7}$: Considering possible future technology upgrades, the design should have a certain degree of scalability to allow for the addition of new functions or improved performance in the future. The corresponding relationship between user requirements and design requirements can be expressed as shown in Figure 7. On the left side, the boxes represent distinct user requirements, such as “monitoring of well conditions without repair” and “high accuracy and reliability”. On the right side, the boxes represent the corresponding design requirements that address these user needs, such as “accurate sonic wave transmission and reception” and “calculation of well temperature and pressure”.

It is important to note that the relationships between the boxes on both the left and right sides are parallel rather than progressive. Each user requirement on the left corresponds to a specific design requirement on the right, and these pairs function independently of the others. There is no hierarchical or sequential dependency between the requirements on the same side; instead, they are addressed simultaneously through distinct solutions, as indicated by the connecting lines in the diagram.

**Figure 8:**
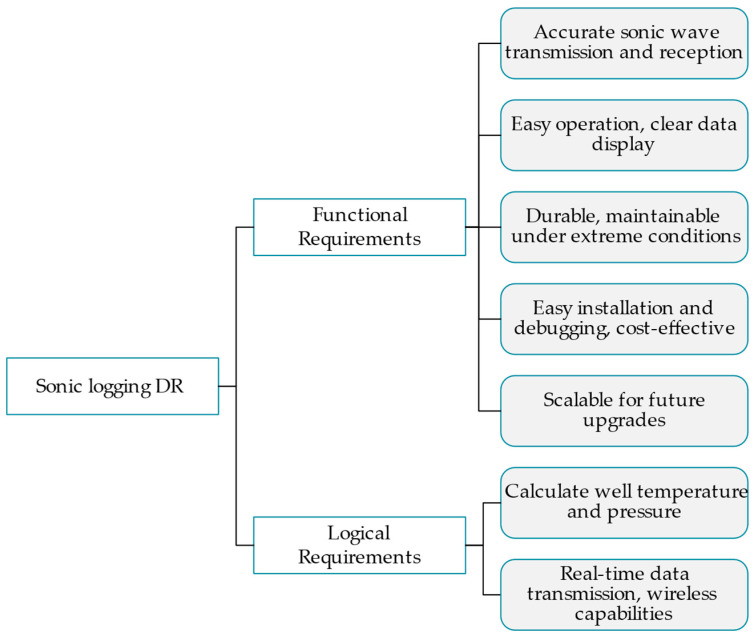
DR hierarchy model of the sonic logging instrument.

Next, the hierarchical model of design requirements is used to classify and sort design requirements and identify those that need to be prioritized during the design process; the result is the hierarchy shown in Figure 8.

To ensure the effective fulfillment of the design requirements, the next step involves using a model-based approach to conceptual design. This approach will help to systematically address the identified requirements and transform them into a coherent multi-perspective design solution. By leveraging model-based techniques, we can enhance the precision and efficiency of the design process, ensuring that all functional and logical requirements are fully satisfied.

### 5.3. Conceptual Design Using Model-Based Approach

**Figure 9:**
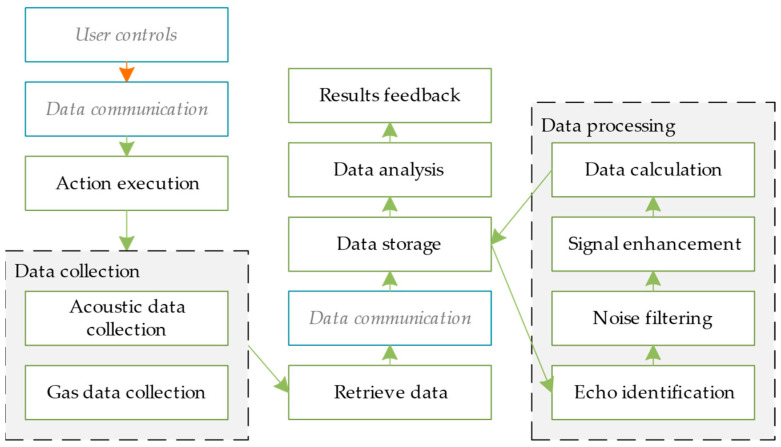
Logic layer design.

The logic layer plays a key role in the design process, implementing the system’s controlling logic and data exchange to ensure the efficient operation of the system. The resultant design of the logic layer is shown in Figure 9. In this figure, the blue boxes represent essential components of the image but are not the focal point of the design; hence, they are displayed in grey font to de-emphasize them. The green arrows indicate the logical design flow, while the orange arrows represent other flows.

**Figure 10:**
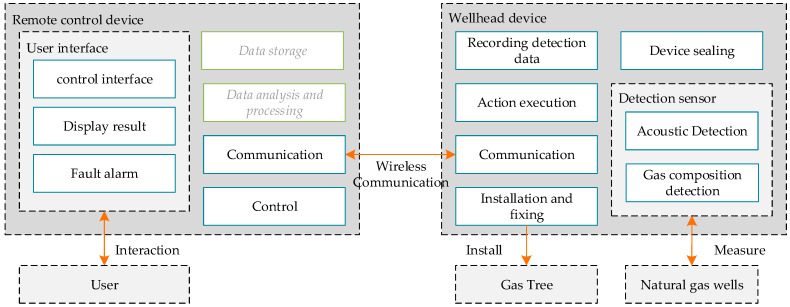
Function design result.

The resulting function layer, as shown in Figure 10, provides a comprehensive view of how these components interact and contribute to the overall functionality of the system. In the diagram, green boxes are used to represent essential parts of the design scheme, which also form part of the outputs from the previous logical design phase. However, these are not the focal points of the diagram; thus, they are displayed in grey font to de-emphasize them.

**Figure 11:**
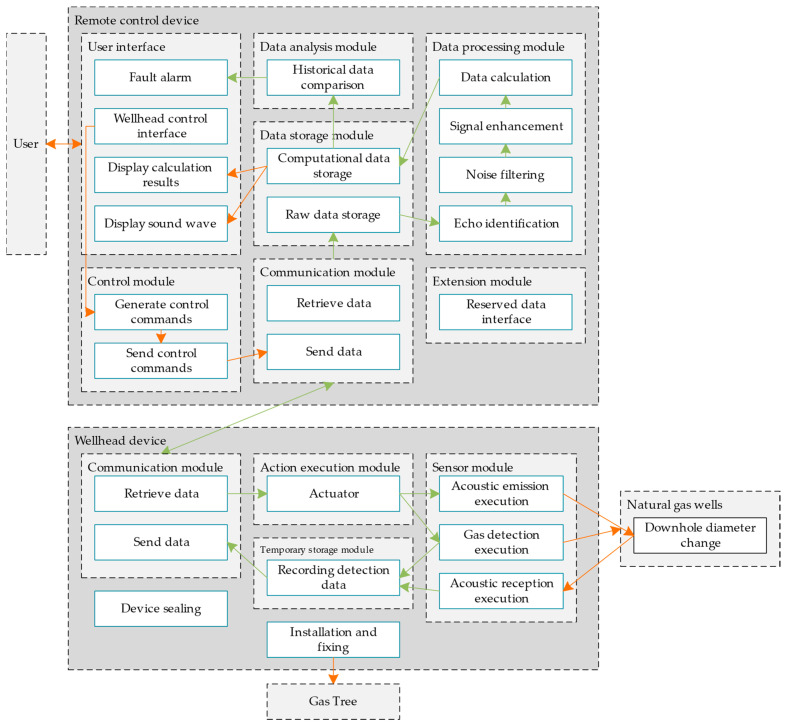
System behavior design result.

The systemic behavioral layer mainly describes the dynamic interaction between and operation of the modules in the system to ensure that the entire system can efficiently and harmoniously complete predetermined tasks; its design is shown in Figure 11. The green line in the figure represents the logical design process, and the orange line represents the design process of other links.

**Figure 12:**
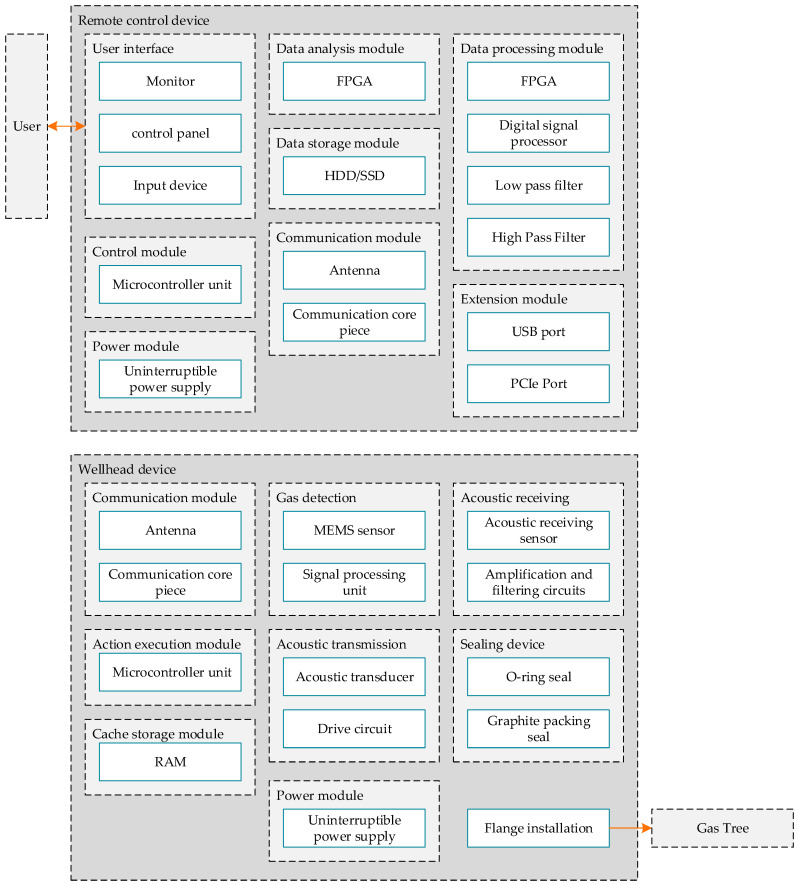
Structural layer design.

The structural layer defines the physical components and hardware configuration of the system, ensuring the installation and coordination of each component. The design result is shown in Figure 12:

### 5.4. Simulation of Prototype Sonic Logging Instrument

**Figure 13:**
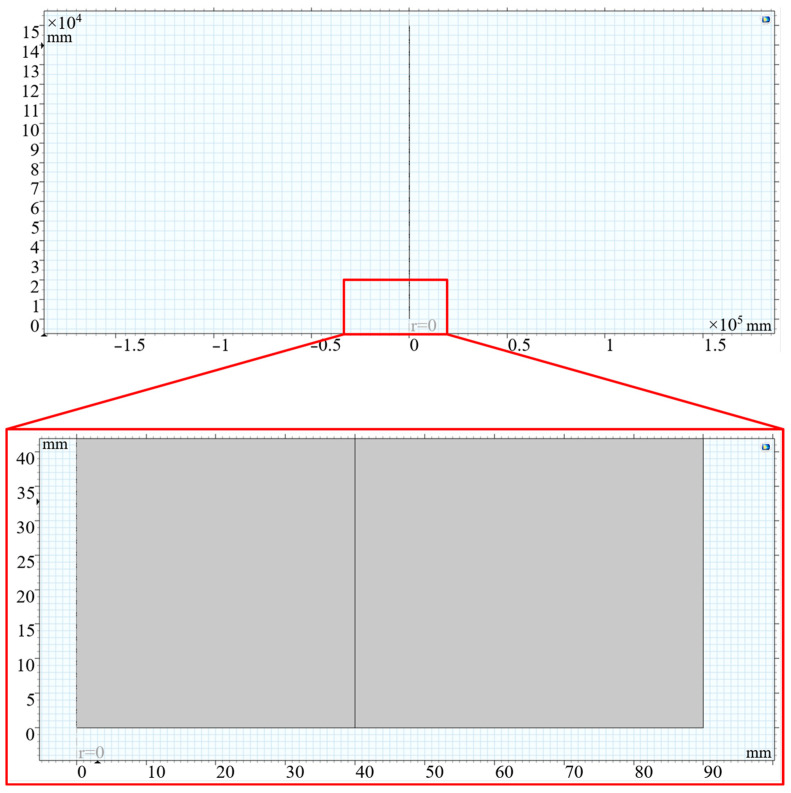
Two-dimensional axisymmetric model of a natural gas well.

In order to verify the effectiveness of the sonic logging instrument, this paper uses COMSOL to perform a finite element simulation of downhole sonic transmission. First, a two-dimensional axisymmetric model of a natural gas well was constructed, as shown in Figure 13. The outer diameter of the oil pipe was set to 180 mm, and the inner diameter was set to 80 mm.

**Figure 14:**
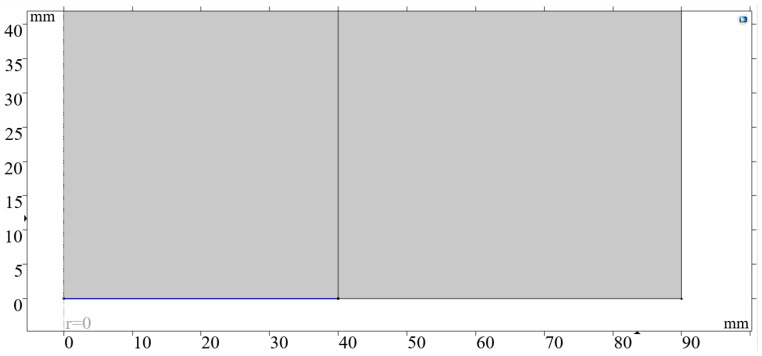
Cylindrical wave radiation initiated inside the oil pipe.

The pipe wall material was cast iron, and the pipe material was air. The pressure acoustics element of COMSOL was selected for simulation using the transient (actd) physics field. As shown in Figure 14, cylindrical wave radiation was initiated in the oil pipe, The sound waves are loaded on the blue lines in the diagram.

The acoustic wave incident pressure field settings are shown in Table 1.

The parameters of the oil pipe mesh were set, as shown in Table 2. The entire model was constructed into a free triangle mesh.

**Figure 15:**
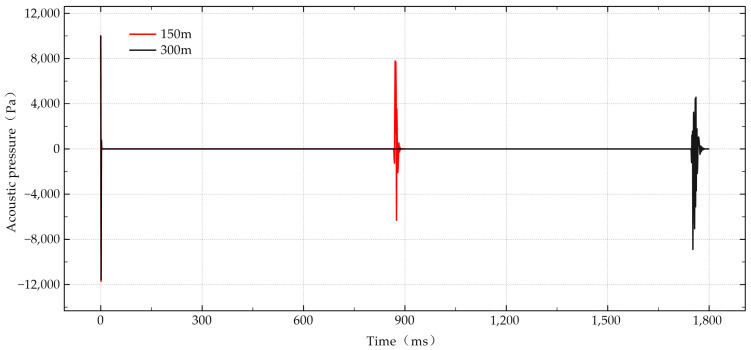
Uniform oil pipe measurement results.

First, a 2 kHz sound wave was used to apply a sound pressure of 10 kPa at the center of the pipe mouth. The ambient temperature and pressure were set to 20 degrees Celsius and one standard atmosphere. Two oil pipes with uniform inner diameters of 150 m and 300 m were selected. After receiving the sound pressure at the center of the pipe mouth and sampling every 1 ms according to time-varying sound pressure, the sound pressure measurement results shown in Figure 15 were obtained.

**Figure 16:**
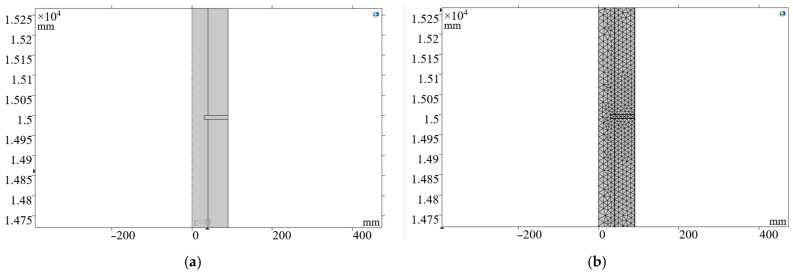
Model setup for simulating sonic reflection points in an oil pipe: (**a**) schematic of variable cross-sections at 15 m intervals; (**b**) refined mesh of variable-diameter sections for enhanced simulation accuracy.

Next, a diameter change was set every 15 m in the 150 m oil pipe, with a thickness of 10 mm and an inner diameter of 60 mm, to simulate the acoustic reflection point in the well. The result is shown in Figure 16a; the mesh was encrypted after the diameter change to enhance the credibility of the simulation results. The pre-processing result is shown in Figure 16b.

**Figure 17:**
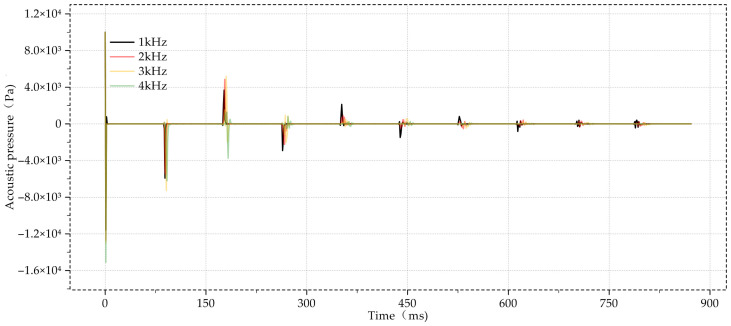
Result of sonic measurement of cross-sectional diameter variations in the wellbore.

Acoustic wave measurement is able to detect any changes in cross-sectional diameter in the wellbore. The expansion of the cross-sectional diameter caused the signal to spike upward to the peak, while the reduction in the cross-sectional diameter caused the signal to fall downward to the trough. By evenly setting the diameter change points in the well, the sound waves with a test frequency of 1 kHz to 4 kHz were tested, and the results are shown in Figure 17.

### 5.5. Discussion

The simulation results demonstrate that, for uniform oil pipes with inner diameters of 150 m and 300 m, the attenuation of sound pressure is not significant, and clear bottom-hole reflection echoes can be received. We observed that lower-frequency sonic waves produce more pronounced downhole echoes. This indicates that the designed sonic logging instrument can effectively transmit and receive sonic signals over long distances without significant signal loss, which is crucial for the accurate monitoring of wells.

Using the uniform oil pipe setup, the 2 kHz sound wave application at the pipe mouth center resulted in consistent sound pressure measurements, highlighting the instrument’s ability to maintain signal integrity in a stable cross-sectional environment. This stability is essential for ensuring that the sonic logging instrument can provide reliable data in standard operating conditions.

When simulating the variable cross-section oil pipe, changes in diameter every 15 m produced distinct reflections, with expansions causing upward signal peaks and contractions causing downward signal troughs. These results confirm the instrument’s sensitivity to structural changes within the wellbore, which enables it to detect and locate anomalies such as blockages or corrosion. The refined mesh in sections of variable diameter further enhanced the accuracy of the simulation, ensuring that the instrument can precisely detect and interpret sonic reflections caused by changes in wellbore geometry.

Additionally, testing with sonic waves at frequencies ranging from 1 kHz to 4 kHz showed that the instrument could detect cross-sectional diameter variations with high resolution. Lower frequencies yielded more pronounced measurement results, suggesting that, for certain applications, optimizing frequency can improve the instrument’s detection capabilities.

## 6. Conclusions

The main contributions and findings of this study are summarized as follows:

- A novel design framework: This study introduces a systematic conceptual design scheme for sonic logging instruments in natural gas wells. The proposed framework integrates a logical layer that operates in parallel with the functional, systemic behavioral, and structural layers, providing a multi-perspective and model-based approach to fulfilling the demands of complex designs;
- Validation through finite element simulations: The feasibility and potential applicability of the proposed design were preliminarily validated using finite element simulation tools. The simulations demonstrated that the designed instrument can effectively transmit and receive sonic signals over long distances with minimal signal loss, particularly for uniform oil pipes with inner diameters of 150 m and 300 m;
- Sensitivity to structural changes: The instrument was shown to be highly sensitive to structural changes within the wellbore, such as diameter variations every 15 m. These changes produced distinct reflection patterns in the simulations, confirming the instrument’s capability to detect and locate anomalies like blockages or corrosion;
- Optimization potential through frequency adjustment: Testing with sonic waves at frequencies ranging from 1 kHz to 4 kHz revealed that the instrument could detect variations in cross-sectional diameter with high accuracy. The results suggest that optimizing frequency, particularly at lower ranges, could further enhance the instrument’s detection capabilities. Future Work with Real Logging Data: While this study has primarily relied on finite element simulations to validate the proposed design, future research will focus on incorporating real logging data to further verify the results. Utilizing real or publicly available logging data will enhance the credibility of the findings, demonstrating the practical applicability of the design method in actual field scenarios. This step will not only strengthen the robustness of the simulation outcomes but also showcase the instrument’s reliability and effectiveness in real-world well monitoring;
- Future work with real logging data: Although this study utilized finite element simulations to validate the proposed design framework, future research will focus on incorporating real logging data to further verify the simulation results. The use of real or publicly available data will provide additional validation, making the findings more robust and convincing. This approach will also demonstrate the practical applicability and reliability of the proposed design method in real-world logging scenarios, strengthening its relevance for field applications.

## Figures and Tables

**Table 1:** Acoustic pressure field parameters.

Element Size Parameter	Value
Temperature	293.15 K
Acoustic pressure	10,000 Pa
Absolute pressure	1 atm
Signal frequency	2 kHz

**Table 2:** Oil pipe element size parameters.

Element Size Parameter	Value
Maximum element size	20 mm
Minimum element size	3 mm
Maximum element growth rate	1.1
Curvature factor	0.2
Resolution of narrow regions	1

## Data Availability

All relevant data are included in this paper.
